# Virtual calcium removal in calcified coronary arteries with photon-counting detector CT—first *in-vivo* experience

**DOI:** 10.3389/fcvm.2024.1367463

**Published:** 2024-02-22

**Authors:** Victor Mergen, Stéphane Rusek, Filippo Civaia, Philippe Rossi, Rengarajan Rajagopal, Eduardo Bättig, Robert Manka, Alessandro Candreva, Matthias Eberhard, Hatem Alkadhi

**Affiliations:** ^1^ Diagnostic and Interventional Radiology, University Hospital Zurich, University of Zurich, Zurich, Switzerland; ^2^ Monaco Cardiothoracic Center, Monaco, Monaco; ^3^ Department of Diagnostic and Interventional Radiology, All India Institute of Medical Sciences, Jodhpur, India; ^4^ Department of Radiology, La Fe University and Polytechnic Hospital, Valencia, Spain; ^5^ Department of Cardiology, University Heart Center, University Hospital Zurich, University of Zurich, Zurich, Switzerland; ^6^ Institute of Radiology, Spitäler fmi AG, Spital Interlaken, Unterseen, Switzerland

**Keywords:** coronary CT angiography (CCTA), coronary artery disease, calcified plaque, photon-counting detector CT (PCD-CT), spectral imaging, virtual non-calcium imaging

## Abstract

**Purpose:**

To evaluate the feasibility and accuracy of quantification of calcified coronary stenoses using virtual non-calcium (VNCa) images in coronary CT angiography (CCTA) with photon-counting detector (PCD) CT compared with quantitative coronary angiography (QCA).

**Materials and methods:**

This retrospective, institutional-review board approved study included consecutive patients with calcified coronary artery plaques undergoing CCTA with PCD-CT and invasive coronary angiography between July and December 2022. Virtual monoenergetic images (VMI) and VNCa images were reconstructed. Diameter stenoses were quantified on VMI and VNCa images by two readers. 3D-QCA served as the standard of reference. Measurements were compared using Bland-Altman analyses, Wilcoxon tests, and intraclass correlation coefficients (ICC).

**Results:**

Thirty patients [mean age, 64 years ± 8 (standard deviation); 26 men] with 81 coronary stenoses from calcified plaques were included. Ten of the 81 stenoses (12%) had to be excluded because of erroneous plaque subtraction on VNCa images. Median diameter stenosis determined on 3D-QCA was 22% (interquartile range, 11%–35%; total range, 4%–88%). As compared with 3D-QCA, VMI overestimated diameter stenoses (mean differences −10%, *p* < .001, ICC: .87 and −7%, *p* < .001, ICC: .84 for reader 1 and 2, respectively), whereas VNCa images showed similar diameter stenoses (mean differences 0%, *p* = .68, ICC: .94 and 1%, *p* = .07, ICC: .93 for reader 1 and 2, respectively).

**Conclusion:**

First experience in mainly minimal to moderate stenoses suggests that virtual calcium removal in CCTA with PCD-CT, when feasible, has the potential to improve the quantification of calcified stenoses.

## Introduction

Coronary computed tomography angiography (CCTA) represents the first-line imaging modality for evaluating coronary artery disease in patients at low to intermediate disease risk (1, 2). In patients with stable chest pain, adding CCTA to conventional testing has shown to reduce the risk of cardiovascular death and future non-fatal myocardial infarction (3). When used instead of invasive coronary angiography, CCTA is associated with a similar long-term major adverse cardiovascular event rate but with a lower rate of major periprocedural complications (4). However, imaging coronary arteries with CCTA may still be inaccurate particularly in patients with severe calcifications resulting in uninterpretable coronary segments and/or overestimation of coronary stenosis due to the effect of calcium blooming (5–7).

The recently introduced dual-source photon-counting detector CT (PCD-CT) system expands the capabilities of CCTA by enabling the imaging of coronary arteries with either the ultra-high-resolution (8–11) or the spectral mode at a maintained high temporal resolution of 66 ms (12–16). The ultra-high-resolution mode has shown to reduce blooming artefacts, to improve plaque and coronary stent lumen visualization (9, 17–19), and to provide high accuracy for the diagnosis of coronary artery disease even in high risk patients (20). However, the ultra-high-resolution mode currently does not allow image acquisition with spectral resolution (9). The scan mode with spectral image acquisition allows dedicated post-processing with separation of iodine in the vessel lumen from calcified vessel wall plaques yielding virtual non-calcium (VNCa) images (13). In a recent phantom experiment using moving vessels filled with iodinated contrast media, this VNCa algorithm indicated reduced blooming artifacts from calcified plaques compared with virtual monoenergetic images (VMI) (13), the latter representing the standard images for diagnostic reading with PCD-CT (21).

The purpose of this study was to evaluate the feasibility and accuracy of quantification of calcified coronary stenoses using VNCa images in CCTA with PCD-CT compared with quantitative coronary angiography (QCA).

## Materials and methods

### Patients

This retrospective single-center study was conducted at a tertiary care cardiovascular medical center after institutional review board and local ethics committee approval (reference number CCM-2022-11-28-074-FC) was obtained. All patients provided written informed consent to allow inclusion of their anonymized data in retrospective analyses.

**Figure 1:**
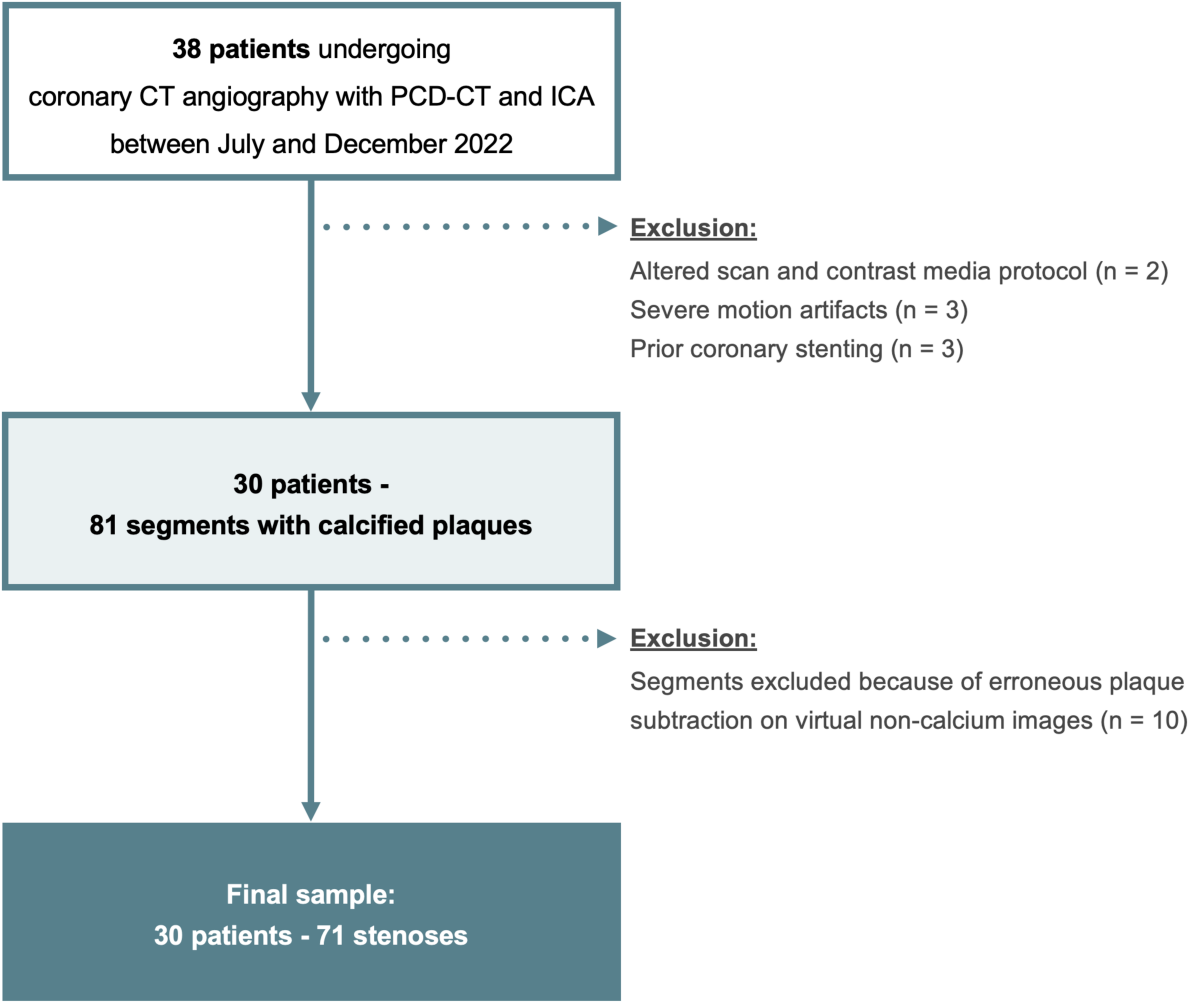
Flowchart of the study. PCD-CT, photon-counting detector CT; ICA, invasive coronary angiography.

Consecutive patients undergoing CCTA with PCD-CT and invasive coronary angiography within 45 days between July and December 2022 for suspected or known coronary artery disease were identified. Exclusion criteria were an extended scan range and altered contrast media protocol, severe motion artifacts because of arrhythmia, lack of coronary calcifications, and coronary stents (Figure 1).

### CT data acquisition and image reconstruction

All scans were performed on a first-generation dual-source PCD-CT system (NAEOTOM Alpha; version syngo CT VA50; Siemens Healthineers AG; Forchheim, Germany) equipped with two cadmium telluride detectors. Patients were pretreated with intravenous beta-blockers for heart rate control if they had an initial heart rate above 70 beats per min (bpm). Sublingual nitroglycerin (2.5 mg isosorbide dinitrate) was administered prior to each scan.

**Figure 2:**
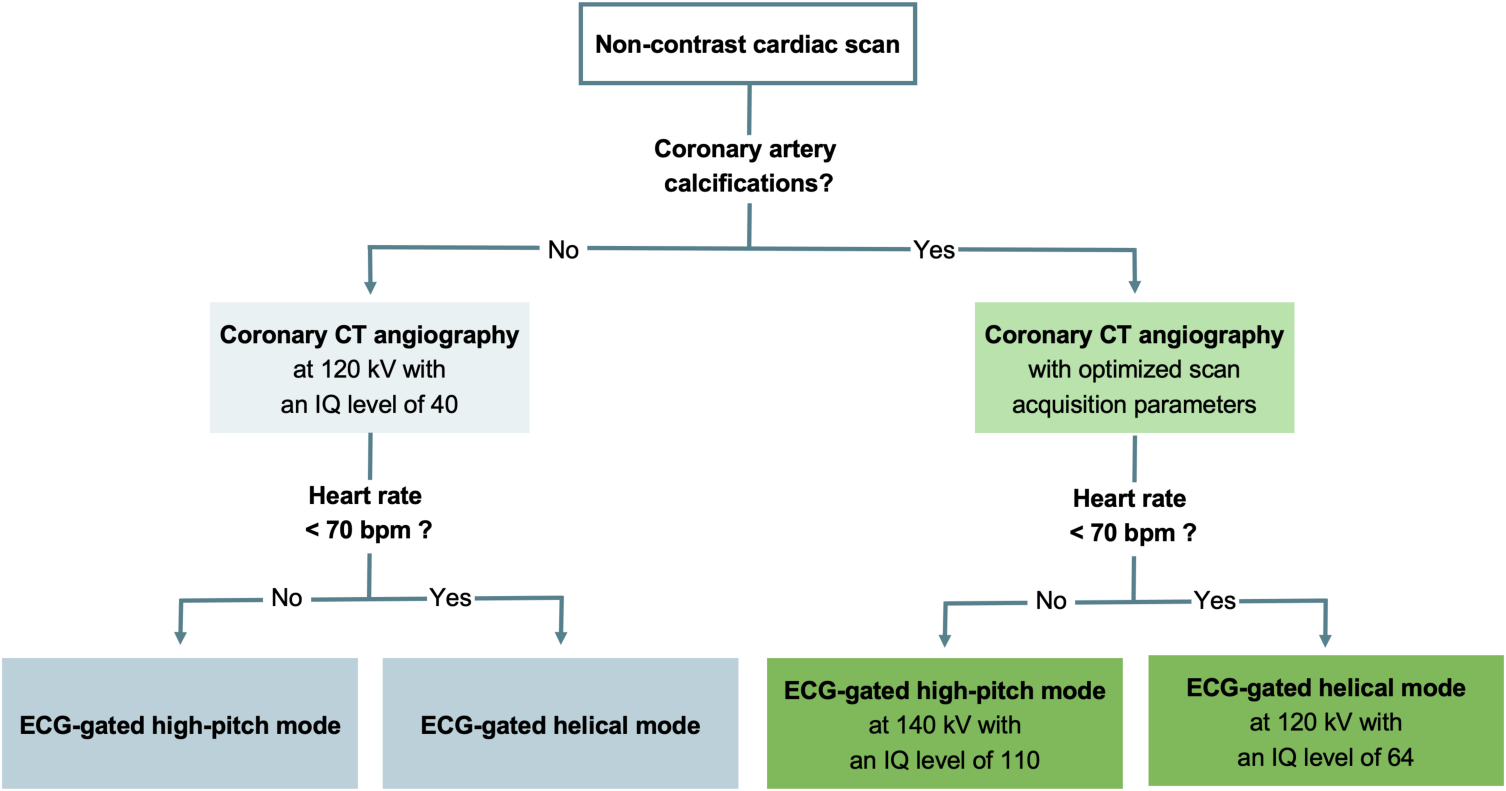
Flowchart detailing the scan protocol. ECG, electrocardiography; IQ, image quality.

The protocol consisted of an electrocardiography (ECG)-gated non-contrast cardiac scan followed by an ECG-gated CCTA acquisition. The non-contrast scan acted as gatekeeper for defining two different CCTA protocols, depending on the presence or absence of coronary artery calcium (Figure 2). In the presence of any coronary artery calcification, scan parameters were further adjusted according to the heart rate. For heart rates below 70 bpm, CCTA was acquired in the ECG-gated high-pitch spectral mode (QuantumPlus) with a pitch factor of 3.2, a tube voltage of 140 kVp, an image quality (IQ) level of 110 using automated tube current modulation (CARE Dose4D, Siemens), and scan acquisition initiated at 65% of the RR-interval. If the heart rate was 70 bpm or higher, CCTA was performed in the retrospective ECG-gated helical spectral mode (QuantumPlus) with a tube voltage of 120 kVp, an IQ level of 60 using automated tube current modulation (CARE Dose4D, Siemens), and ECG-pulsing from 65% to 75% of the RR-interval. In the absence of coronary artery calcium, CCTA was performed with a tube voltage of 120 kV and an IQ level lowered to 40 to optimize the radiation dose. Detector collimation was 144 × 0.4 mm and the gantry rotation time was 0.25 s for all scans. CCTA was initiated after the injection of a weight-based volume of iodinated contrast medium (60–80 ml, iomeprole, Iomeron 350 mg I/ml; Bracco Imaging France, Massy, France) followed by a saline chaser (50 ml, NaCl 0.9%).

CCTA images were reconstructed as vascular spectral post-processed (VSPP) images using a vascular kernel (Bv44) to compute VMI and a quantitative kernel (Qr44) to compute optimized VNCa images (PURELumen, Siemens), as previously recommended (13). VMI were reconstructed at 70 keV (22) with a slice thickness of 0.4 mm, an increment of 0.2 mm, and using quantum iterative reconstruction (QIR) at a level of 4. VNCa images were reconstructed with a slice thickness of 0.6 mm, an increment of 0.4 mm, and a QIR level of 3 as previously suggested (13). The field of view was 180 × 180 mm^2^ and the matrix size was 512 × 512 pixels for both reconstructions.

### Stenosis quantification

Two independent readers (V.M., in-training with 4 years of experience, and S.R., with 28 years of experience in cardiovascular imaging) performed stenosis quantification from calcified plaques on CCTA and were blinded to the QCA results. One reader (V.M.) assessed in which coronary segments stenoses were localized (23) and shared these segments with the second reader (S.R.). Any stenosis was considered, even if not significant, defined as a percentage diameter stenosis of less than 50%. If more than one stenosis from calcified plaques was present in a coronary segment, the most severe stenosis was assessed. This reader (V.M.) also evaluated VNCa images for potential artifacts due to erroneous plaque subtraction and excluded these segments from further evaluation. Plaque subtraction was considered erroneous if parts of the contrast-enhanced coronary lumen were subtracted together with the calcified plaque, using VMI as reference. Coronary stenoses were then quantified according to a previously described method (19), first on VMI and second on VNCa images. Quantification of CCTA images was performed with a window level and width of 300 HU and 800 HU, respectively, as previously suggested (24).

ICA was performed by board-certified cardiologists in accordance with current guidelines (25). Each vessel was evaluated by at least two different projections. A third independent and blinded reader (R.R., board-certified radiologist with 7 years of experience) assessed the stenoses with three-dimensional (3D) QCA using dedicated software (CAAS Workstation, Version 8.5.2., Pie Medical Imaging, Maastricht, the Netherlands). The software first reconstructs three-dimensional images from at least two end-diastolic projections separated by more than 30°. Then, the software's semi-automatic lumen contour detection allows measurement of diameter stenoses, taking into account the vessel portions that are 20 mm proximal and 20 mm distal to the minimum lumen diameter. 3D-QCA measurements in the first five patients were supervised by a fourth experienced reader (A.C., board-certified cardiologist with 5 years of experience in ICA]. 3D-QCA measurements served as the reference standard.

Diameter stenoses were also classified according to the Coronary Artery Disease-Reporting and Data System (CAD-RADS) (23), where 0% corresponded to no stenosis, 1%–24% to minimal stenosis, 25%–49% to mild stenosis, 50%–69% to moderate stenosis, 70%–99% to severe stenosis, and 100% to occlusion. Readers were blinded to the patient's medical history and symptoms, and to stenosis quantification results from the other readers.

### Statistical analysis

Analyses were performed using R statistical software (R, version 4.3.0; R Foundation for Statistical Computing, Vienna, Austria, https://www.R-project.org/). Variables are presented as mean ± standard deviation and as median and interquartile range depending on their distribution. Categorical variables are reported as counts and percentages. Stenosis quantifications determined on VMI and VNCa images were compared with the reference standard QCA using Bland–Altman analyses and Wilcoxon signed-rank test. Inter-reader agreement of stenosis quantification was assessed using two-way intraclass correlation (ICC) with 0.61–0.80 indicating substantial agreement and 0.81–1.00 indicating excellent agreement (26). Agreement of stenoses categories determined using VMI and VNCa images, respectively, and 3D-QCA were assessed by weighted Kappa analysis. *P*-values were adjusted with the Benjamini–Hochberg procedure for multiple comparisons. A two-tailed *P*-value < .05 was considered to infer statistical significance.

## Results

### Patient characteristics

Two of the 38 patients were excluded because of an altered scan and contrast media protocol, three patients because of severe motion artifacts, and three patients were excluded because of coronary stents. In the 30 remaining patients, there were 81 stenoses from calcified plaques. Ten of 81 (12%) stenoses were excluded because of erroneous plaque subtraction on VNCa images (Supplementary Table S1). Finally, 30 patients (4 women, 26 men; mean age, 64 ± 8 years) were included and 71 stenoses evaluated (Figure 1). Patient demographics and distribution of coronary stenoses is shown in Tables 1, 2.

**Table 1:** Patient demographics.

	All patients (*n* = 30)
Patient characteristics	
Sex	
Male	26/30 (87)
Female	4/30 (13)
Age [years]	64 ± 8 (range, 47–79)
Body mass index [kg/m^2^]	26.4 ± 6.1 (range, 17.3–49.3)
Heart rate during data acquisition [bpm]	60 ± 7 (range, 50–80)
Coronary artery calcium score^a^	177 (interquartile range, 121–361)
Medical history	
Arterial hypertension	7/30 (23)
Dyslipidemia	16/30 (53)
Diabetes	4/30 (13)
Smoking	14/30 (47)
Radiation dose estimates of coronary CT angiography	
ECG-gated high-pitch mode (*n* = 19)	
Volume CT dose index [mGy]	4.6 ± 0.8
Dose length product [mGy · cm]	101 ± 20
ECG-gated retrospective helical mode (*n* = 11)	
Volume CT dose index [mGy]	16.8 ± 6.7
Dose length product [mGy · cm]	357 ± 114

Unless otherwise specified, data are mean ± standard deviation or proportion of patients (percentage).

bpm, beats per minute; ECG, electrocardiography.

^a^ Data are median and interquartile range in parentheses. The coronary artery calcium score was determined on non-contrast images.

**Table 2:** Coronary artery segments with calcified plaques.

Coronary artery segment	Number of assessed segments (*n* = 71)
Right coronary artery (RCA)	
Proximal (segment 1)	8
Middle (segment 2)	6
Posterior descending artery (segment 4)	3
Posterolateral branch from RCA (segment 16)	1
Left main coronary artery (LM) (segment 5)	3
Left anterior descending artery (LAD)	
Proximal (segment 6)	17
Middle (segment 7)	17
Apical (segment 8)	1
First diagonal branch (segment 9)	3
Circumflex artery (CX)	
Proximal (segment 11)	5
First obtuse marginal branch (segment 12)	4
Middle and distal (segment 13)	3

Data are number of segments.

### Stenosis quantification

3D-QCA was feasible in all stenoses and revealed a median diameter stenosis of 22% [interquartile range (IQR), 11%–35%; total range, 4%–88%].

On CCTA, the first reader measured a median diameter stenosis of 34% (IQR, 19%–48%; total range, 4%–92%) for VMI and 25% (IQR, 13%–33%; total range, 0%–91%) for VNCa images. Diameter stenoses were overestimated with a mean difference of −10% (limits of agreement, −29%/9%; *p* < .001) for VMI and were similar with a mean difference of 0% (limits of agreement, −12%/12%; *p* = .68) for VNCa images when compared with 3D-QCA.

**Figure 3:**
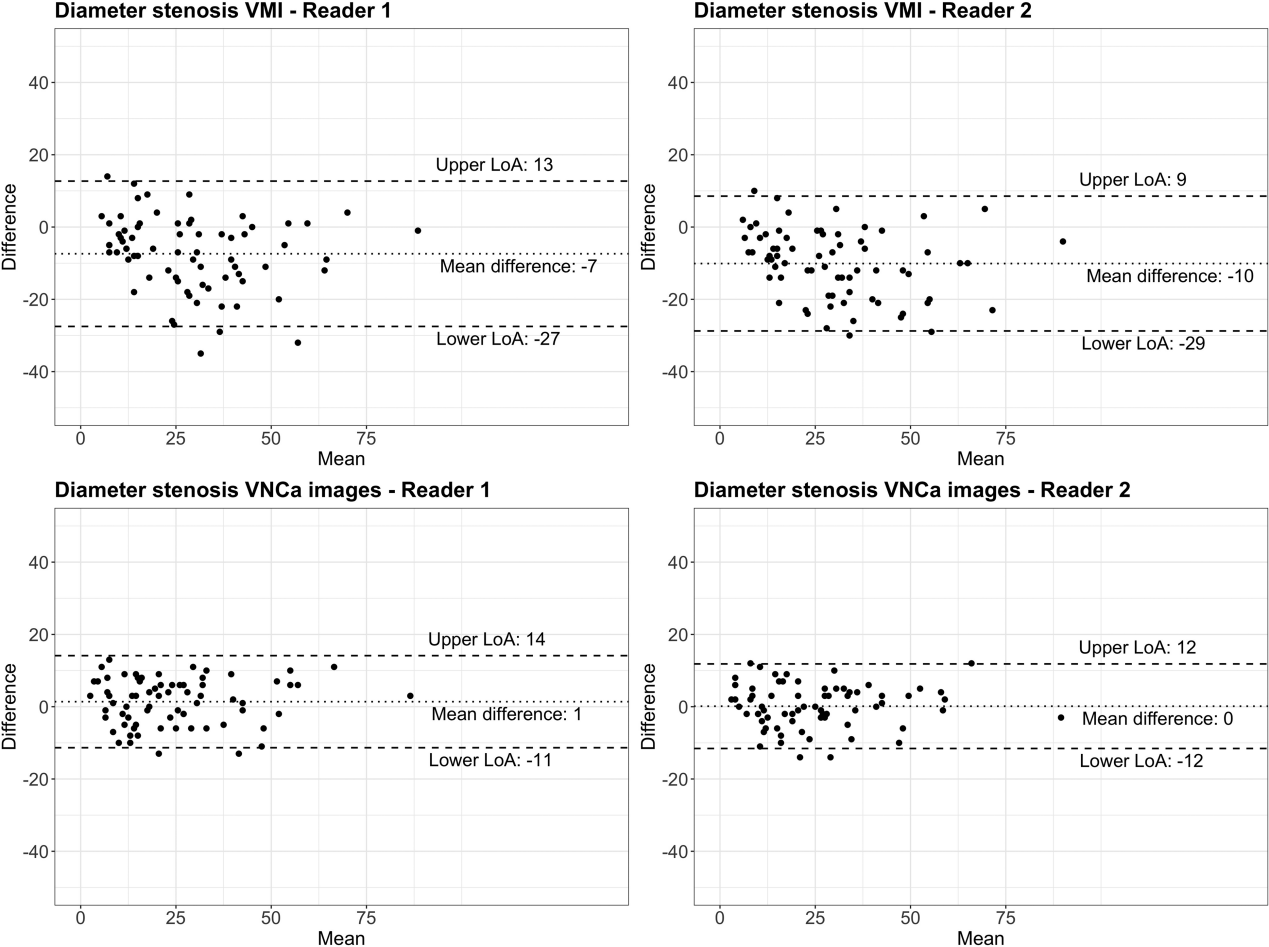
Bland–Altman plots comparing diameter stenosis determined by quantitative coronary angiography (reference standard) with coronary CT angiography using conventional virtual monoenergetic images (VMI) and virtual non-calcium (VNCa) images for reader 1 and reader 2, respectively. The dotted line indicates the mean difference; the dashed lines indicate the upper and lower limits of agreement (LoA).

On CCTA, the second reader measured a median diameter stenosis of 32% (IQR, 15%–45%, total range, 0%–89%) for VMI and 19% (IQR, 12%–30%; total range, 0%–85%) for VNCa images. When compared with 3D-QCA, diameter stenoses were overestimated with a mean difference of −7% (limits of agreement, −27%/13%; *p* < .001) for VMI, whereas VNCa images revealed similar diameter stenoses with a mean difference of 1% (limits of agreement, −11%/14%; *p* = .07) (Figure 3).

**Figure 4:**
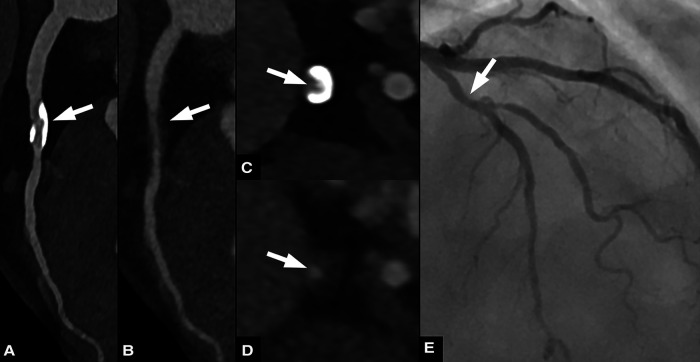
Sixty three-year-old male patient with chronic coronary syndrome. Curved planar reformations of conventional (**A**) and virtual non-calcium (VNCa) images (**B**) show calcified and subtracted calcified plaques, respectively, in the proximal left anterior descending artery (LAD). Corresponding axial images show the calcified plaque (**C**) and the vessel lumen after subtraction (arrow) (**D**) invasive coronary angiography (**E**) confirmed the presence of a moderate stenosis in the proximal LAD.

**Figure 5:**
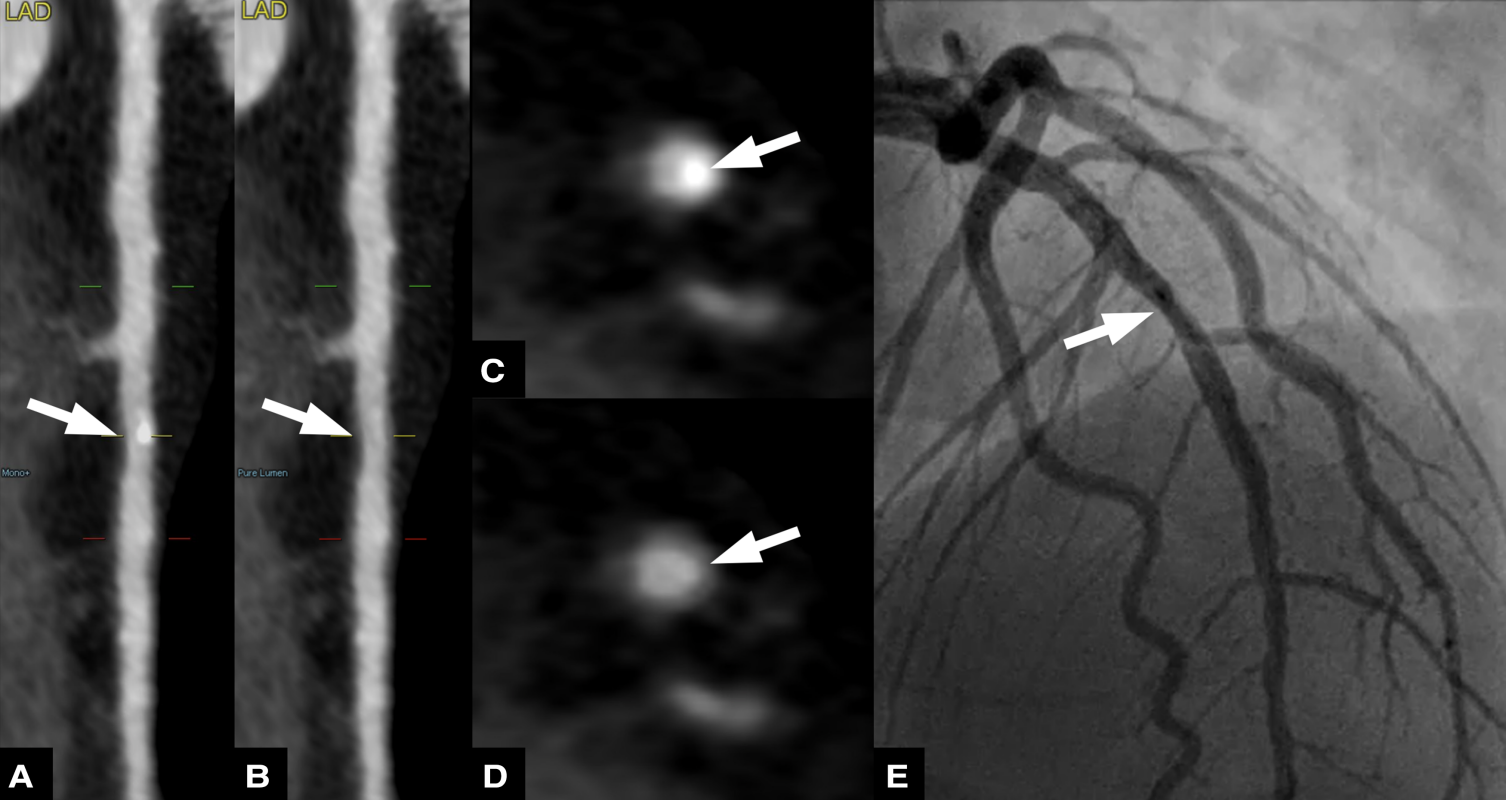
Fifty nine-year-old male patient with suspected coronary artery disease. Straightened multiplanar reformations of conventional (A) and virtual non-calcium (VNCa) images (B) show calcified and subtracted calcified plaques, respectively, in the mid left anterior descending artery (LAD). Corresponding axial images show the calcified plaque (C) and the vessel lumen after subtraction (arrow) (D) invasive coronary angiography (E) confirmed the presence of a mild stenosis in the proximal LAD.

For both readers, agreement of stenosis quantification was higher between VNCa images and 3D-QCA (ICC: .94 and .93 for reader 1 and 2, respectively) than between VMI and 3D-QCA (ICC: .87 and .84 for reader 1 and 2, respectively). Figures 4, 5 provide representatives examples of VMI and VNCa images and the reference standard QCA.

### Stenosis classification

**Figure 6:**
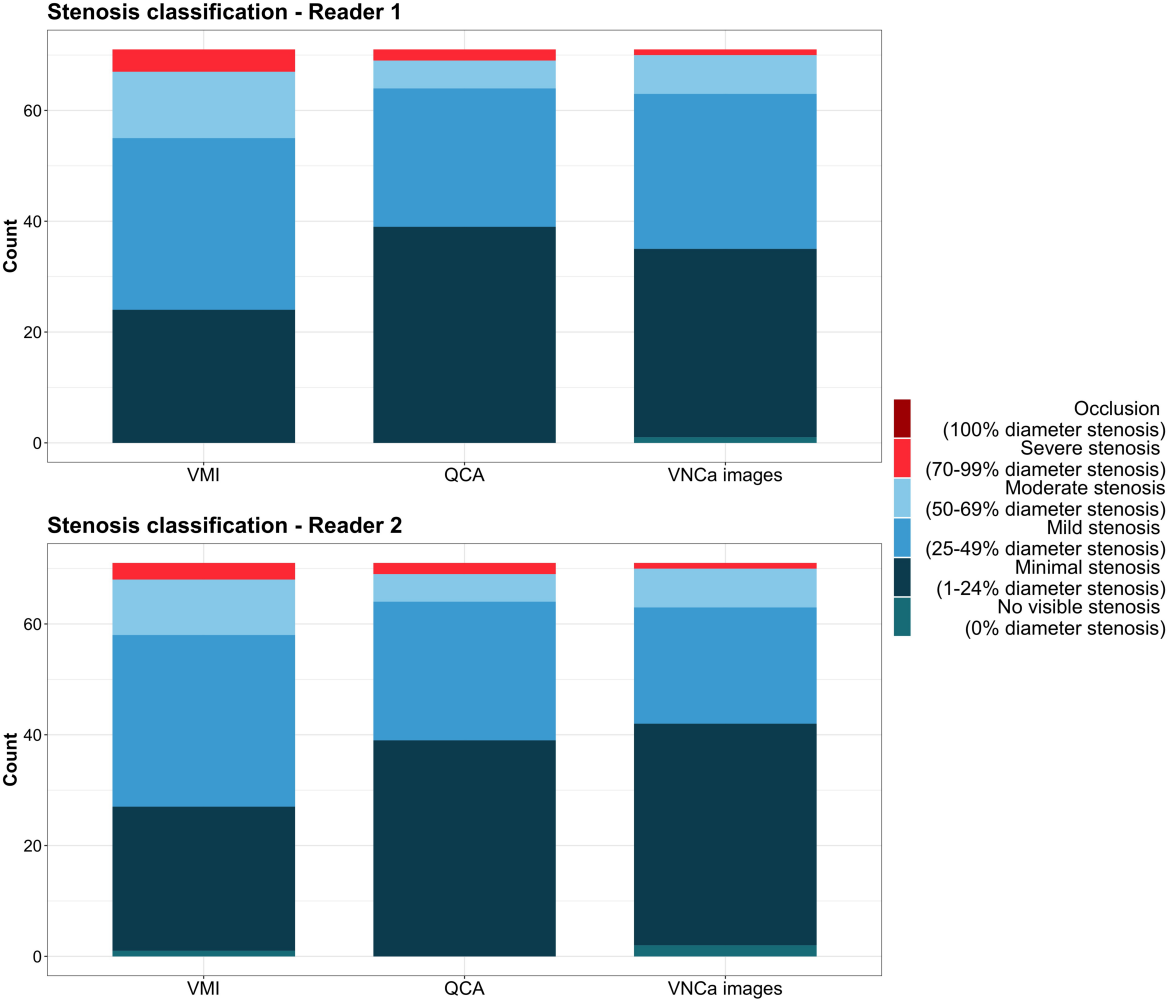
Diagram illustrating the concordance and discordance of stenosis classification according to CAD-RADS determined on quantitative coronary angiography (reference standard), virtual monoenergetic, and virtual non-calcium images. QCA, quantitative coronary angiography; VMI, virtual monoenergetic images; VNCa, virtual non-calcium.

Based on 3D-QCA, there were 39 minimal, 25 mild, five moderate, and two severe stenoses according to the CAD-RADS classification (Figure 6). The concordance of stenosis categories determined on QCA and VMI was 62% (44/71 stenoses, *κ *= .70) and 69% (49/71 stenoses, *κ *= .70) and improved to 87% (62/71 stenoses, *κ *= .89) and 79% (56/71 stenoses, *κ *= .82) when using VNCa images for reader 1 and 2, respectively. When quantifying stenosis on VMI, 37% (26/71) and 27% (19/71) of stenoses were overestimated, while only 8% (6/71) and 7% (5/71) of stenoses were overestimated using VNCa images for reader 1 and 2, respectively.

## Discussion

PCD-CT allows for CCTA with spectral information enabling the spectral based reconstruction of VNCa images. This study reports the initial patient experience with the VNCa algorithm for quantifying calcified coronary stenoses with PCD-CT. The virtual removal of calcium from the iodinated coronary vessel lumen with VNCa was feasible in 88% of the stenoses. As compared with the reference standard 3D-QCA, diameter stenosis quantification was similar using VNCa images, while the conventional assessment yielded an overestimation of stenosis grading. In regard to stenosis categorization, conventional images led to an overestimation in 37% and 27% of calcified coronary stenoses, whereas with VNCa images only 8% and 7% of stenoses were overestimated.

In dual-source PCD-CT, vascular images for spectral post-processing are reconstructed as so-called *vascular spectral post-processed* (VSPP) images (27). VSPP images preserve information from the different energy bins and allow computation of VMI from 40 to 190 keV as well as the reconstruction of iodine maps and virtual non-contrast images using dedicated post-processing software. The “vascular” attribute in VSPP accounts for additional spectral information about calcium and iodine separation that is preserved in VSPP images and allows for virtual calcium removal.

The underlying dual energy algorithm of VNCa was initially described by Mannil et al. (28) and was later further developed for PCD-CT (13). This optimized virtual calcium removal algorithm aims to reconstruct images without calcium contribution while preserving all other material, in particular iodine. In brief, the algorithm performs a series of material decomposition into different base materials, synthesizes a monoenergetic image, a non-calcium image, as well as a calcium map, calculates a soft tissue offset and finally blends the monoenergetic image and the iodine image using the calcium map as a weighting image (13). Because VSPP can be reconstructed with different kernels and quantitative iterative reconstruction strengths, care must be taken to select a reconstruction kernel tailored to the diagnostic task. We chose to reconstruct VSPP for visual assessment of coronary stenoses with a vascular kernel (Bv44), which is characterized by edge enhancement and facilitates visual interpretation. VSPP used to compute VNCa images were reconstructed with a quantitative kernel (Qr44), as this kernel was designed for optimal performance when using spectral post-processing applications and quantitative tasks.

Robust performance of dual energy-based post-processing algorithms rely on a high image quality. For CCTA, this means avoiding motion artifacts and applying a sufficiently high IQ level during scan acquisition to achieve a high contrast-to-noise ratio. In general, a broader x-ray spectrum improves spectral separation, which is beneficial for dual energy-based post-processing. Therefore, in this study, patients were pretreated with beta-blockers to ensure a sufficiently low and stable heart rate (average heart rate 60 ± 7 bpm) and with sublingual nitroglycerin for coronary vasodilatation. When the patients' heart rate was sufficiently low (<70 bpm), CCTA was acquired in the ECG-gated high-pitch mode using a tube voltage of 140 kVp and an IQ level of 110. Low and regular heart rates are critical during CCTA acquisition in ECG-guided high-pitch mode, as scan acquisition occurs during a single cardiac cycle and deviations can lead to incorrect timing of the scan and non-diagnostic image quality (29). At higher heart rates (≥70 bpm), CCTA were acquired in the ECG-gated retrospective helical mode with a tube voltage of 120 kV and an IQ level of 64. Radiation doses were approximately threefold when CCTA was acquired in the helical (mean DLP of 357 mGy · cm) compared with the high-pitch mode (mean DLP of 101 mGy · cm). In comparison with the literature, radiation doses of CCTA in the high-pitch mode were higher than those reported for conventional third-generation dual-source energy-integrating detector CT using the same scan mode (DLP 23–46 mGy · cm (30–33) and similar for the helical mode [DLP 340–471 mGy · cm (31, 32)].

Applying a strict scan protocol aiming at a high image quality resulted in exclusion of 12% calcified coronary stenoses due to erroneous plaque subtraction on VNCa images. From these, four were in the group of patients scanned in the retrospective ECG-gated and six in the high-pitch mode. This slight tendency for artifacts towards CCTA data acquired in high-pitch mode merits further evaluation in future studies.

Our results confirm the known issue (5, 6, 34) of overestimation of coronary stenoses in conventional CCTA images (by 10% and 7%, respectively, in our study). In a recent patient study, Vattay et al. demonstrated a significant impact of virtual monoenergetic levels on plaque component volumes (35). In another study, Wolf et al. evaluated the influence of virtual monoenergetic levels on coronary artery stenosis quantification using a PCD-CT in comparison with invasive coronary angiography (34). The authors observed that, even when choosing an optimal monoenergetic level of 100 keV for calcified stenoses, an average overestimation of 17% occurred. In distinction, diameter stenoses on VNCa images were similar to the reference standard (mean differences of 0% and 1%, respectively). These results are in line with those of a previous phantom study using the same VNCa algorithm in moving, contrast-filled vessels with calcified plaques (13). The more accurate stenosis measurements on VNCa images may be attributable to an effective reduction of calcium blooming caused by calcified plaques owing to the advanced dual energy-based iodine-calcium separation. Stenosis classification according to CAD-RADS was also better with VNCa images (concordance of 92% and 93%, respectively), whereas reading on conventional images resulted in an overestimation of stenosis classification of 33% and 27%, respectively.

Based on our preliminary study results, we believe that coronary stenoses assessment should always be assessed *first* on conventional images and *second*—when blooming artifacts from calcified plaques are assumed—on VNCa images. As already previously suggested (13), VNCa images should not be evaluated in isolation because erroneous plaque subtraction could lead to false diagnoses.

This study has the following limitations. First, this single-center retrospective study included a limited number of patients and stenoses, which hindered an in-depth analysis of potential reasons for erroneous plaque subtraction. Second, the tube potentials varied for the different CCTA scan modes, potentially influencing spectral separation. Third, performance of the VNCa algorithm was not evaluated using CCTA acquired with the prospective ECG-gated sequential mode. Fourth, stenosis assessment on VMI and VNCa images was performed sequentially rather than in random order. However, as mentioned above we believe that the VNCa algorithm should be used always in adjunct to conventional coronary artery analysis. Fifth, for a thorough evaluation of the VNCa algorithm, every stenosis was assessed for each patient, regardless of the significance. Although stenoses ranged from 4% to 88%, the majority were in the minimal to mild category. Sixth, scan protocol, reconstruction kernels, slice thicknesses, virtual monoenergetic levels, and QIR strengths were selected based on personal experience, literature (13, 22), and manufacturer recommendations but were not systematically evaluated. Seventh, the inter-reader agreement of QCA was not analyzed. Finally, scan acquisition and spectral applications of PCD-CT continue to evolve. New software versions (Syngo CT VA50 SP1 or VB10) have been released in the meantime that may further improve spectral imaging.

In conclusion, our preliminary experience in mainly minimal to moderate coronary stenoses suggests that virtual calcium removal in coronary CT angiography with photon-counting detector CT, when feasible, has the potential to improve quantification of coronary stenoses. Further optimization of the VNCa algorithm is necessary to reduce erroneous calcium removal for enabling routine clinical application of the technique.

## Data Availability

The raw data supporting the conclusions of this article will be made available by the authors, without undue reservation.
