# Supplementary material for: Virtual calcium removal in calcified coronary arteries with photon-counting detector CT—first in-vivo experience
Source: Front Cardiovasc Med. 2024 Feb 22;11:1367463. doi: 10.3389/fcvm.2024.1367463 (PMC10917906; doi:10.3389/fcvm.2024.1367463)
Supplement: Supplementary file 1 [file Table1.docx]

Supplementary Material

**Supplementary Table 1:** Details about the coronary artery segments excluded because of erroneous plaque subtraction on VNCa images.

| **Patient demographics** | **n=7 patients** |
| --- | --- |
| Sex |  |
| Male | 7/7 (100) |
| Age [years]* | 63 ± 8 (range, 51 – 71) |
| Body mass index [kg/m^2^]* | 26.2 ± 3.0 (range, 23.4 – 31.2) |
| Body mass index >25 kg/m^2^ | 3/7 (43) |
| Body mass index <25 kg/m^2^ | 4/7 (57) |
| Heart rate during data acquisition [bpm]* | 57 ± 4 (range, 50 – 62) |
| **Scan mode** |  |
| ECG-gated retrospective helical mode^†^ | 2/7 (29) patients 4/10 (40) segments |
| >1 excluded segment per patient | 1/7 (14) patient |
| ECG-gated high-pitch mode^†^ | 5/7 (71) patients |
|  | 6/10 (60) segments |
| >1 excluded segment per patient | 1/7 (14) patient |
| **Coronary artery segment** | n=10 excluded coronary artery segments |
| Right coronary artery (RCA) |  |
| Proximal (segment 1) | 2/10 (20) |
| Distal (segment 3) | 1/10 (10) |
| Posterolateral branch from RCA (segment 16) | 2/10 (20) |
| Left anterior descending artery (LAD) |  |
| Proximal (segment 6) | 1/10 (10) |
| First diagonal branch (segment 9) | 1/10 (10) |
| Circumflex artery (CX) |  |
| Middle and distal (segment 13) | 2/10 (20) |
| Posterior descending artery (segment 15) | 1/10 (10) |

Unless otherwise stated, data are numbers of participants, with percentages in parentheses.
bpm = beats per minute. * Data are means ± SDs.
† Number of patients and respective coronary segments with the respective percentage in parentheses excluded due to erroneous plaque subtraction listed according to the acquisition mode of CT coronary angiography
